# The pancreatic tumor microenvironment drives changes in miRNA expression that promote cytokine production and inhibit migration by the tumor associated stroma

**DOI:** 10.18632/oncotarget.10722

**Published:** 2016-07-20

**Authors:** Song Han, David H. Gonzalo, Michael Feely, Daniel Delitto, Kevin E. Behrns, Mark Beveridge, DongYu Zhang, Ryan Thomas, Jose G. Trevino, Thomas D. Schmittgen, Steven J. Hughes

**Affiliations:** ^1^ Department of Surgery, University of Florida, Gainesville, FL 32610, USA; ^2^ Department of Pathology, College of Medicine, University of Florida, Gainesville, FL 32610, USA; ^3^ College of Pharmacy, University of Florida, Gainesville, FL 32610, USA

**Keywords:** miR-200, miR-205, tumor-associated stroma, pancreatic cancer, tumor microenvrionment

## Abstract

The pancreatic adenocarcinoma (PDAC) microenvironment is largely comprised of fibrotic tumor associated stroma (TAS) that contributes to the lethal biology of PDAC. microRNA (miRNA) are small non-coding RNAs that regulate gene expression. We hypothesized that interactions between PDAC cells and TAS cells within the microenvironment modulate miRNA expression and thus, tumor biology. We observed that miR-205 and members of the miR-200 family (miR-200a, -200b, -200c, -141 and miR-429) were exclusively expressed in PDAC cells, consistent with an epithelial miRNA signature, while miR-145 and miR-199 family members (miR-199a and -199b) were solely expressed in TAS cells, consistent with a stromal miRNA signature. This finding was confirmed by qRT-PCR of RNA obtained by laser-capture microdissection of surgical specimens. Using an *in vitro* co-culture model, we further demonstrated regulation of miRNA expression by cell-cell contact. Forced expression in TAS cells of miR-200b/-200c and miR-205 to mimic these observed changes in miRNA concentrations induced secretion of GM-CSF and IP10, and notably inhibited migration. These data suggest interactions within the tumor microenvironment alter miRNA expression, which in turn have a functional impact on TAS.

## INTRODUCTION

Pancreatic ductal adenocarcinoma (PDAC) is a lethal malignancy with 5-year survival rates of roughly 8% [1]. Perhaps the most distinguishing histological characteristic of PDAC is the development of a fibrotic tumor-associated stroma (TAS) that integrates into the malignant epithelial compartment [2]. Numerous reports support the notion that TAS strongly contributes to the malignant phenotype of PDAC. TAS participates in critical paracrine signaling loops that promote PDAC cell survival, therapeutic resistance and metastasis [3]. TAS also produces the dense extracellular matrix characteristic of PDAC, acting as a physical obstruction to infiltrating immune elements and the diffusion of chemotherapies [4, 5]. However, TAS depletion strategies were unsuccessful in clinical trials and may even accelerate PDAC progression in pre-clinical models [6]. Thus, the impact of TAS on PDAC within the tumor microenvironment remains controversial.

In other epithelial malignancies, TAS gene expression strongly affects tumor biology and associates with clinical outcomes [7, 8]. For example, a stroma-derived prognostic predictor (SDPP) based on TAS gene expression patterns improves molecular classification and outcome prediction in breast cancer [7]. In colorectal cancer, TAS contributes the majority of the transcriptome, and prognostic predictive power arises from genes expressed by stromal rather than epithelial cells [8]. While TAS represents the bulk of most PDACs, there has been an absence of investigation in PDAC until very recently. Moffitt and colleagues identified similar gene expression patterns that classified PDAC into tumor-specific (basal-like) and stromal-specific subtypes with prognostic relevance; a stromal-specific subtype was related to reduced survival [9]. These studies emphasize that gene expression analyses that evaluate whole tissues frequently lack the ability to discern potentially important differences in gene expression that are specific to the epithelial versus stromal compartments of a given malignancy.

This concept may also be true for microRNA (miRNA) - endogenous epigenetic gene expression regulators. Differential expression of miRNAs between the epithelial and stromal cells has been identified in colorectal tissues [10] and early breast lesions [11]. A considerable effort has been invested in identifying miRNA as biomarkers for pancreatic cancer [12–15], but these have been assessments of whole tissue nucleic acid isolates or blood/serum from patients. Aberrant miRNA expression associates with PDAC proliferation, invasion, treatment resistance and poor prognosis [16–18]. However, differential expression of miRNA between TAS and PDAC and the potential role of miRNA in stromal evolution and epithelial-stromal interplay in PDAC remains unknown. Thus, we aimed to identify miRNA expression signatures specific to the epithelial or stromal compartment in PDAC, and subsequently test the hypothesis that interactions within the tumor microenvironment impact microRNA expression and thus, tumor biology.

## RESULTS

### miRNA expression differs between cultured PC and TAS cells

**Figure 1:**
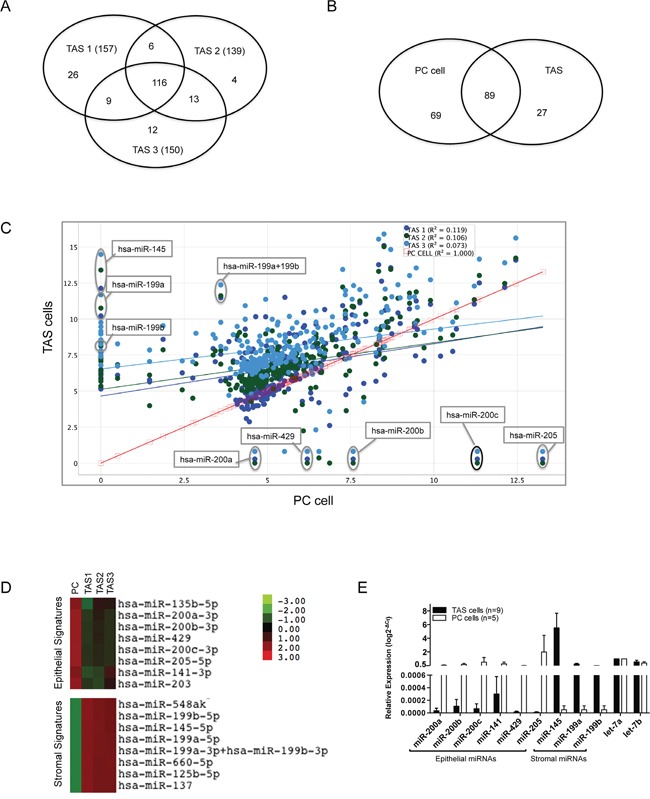
Differential miRNA expression of PC cells and TAS cells **A**. Venn diagram demonstrating large portions of overlapped miRNA probes detected among three TAS cell lines TAS1, TAS2 and TAS3. **B**. Venn diagram summarizing the miRNA probes that were overlapping or different between TAS cells (n=3) and the representative PC cells. **C**. Scatter plot of differentially expressed miRNAs in TAS cells at y-axis (n=3, dots in cold colors) vs. PC cells at x-axis (square in red). Data are plotted as log2 digital signal readings. *r^2^*: coefficient of determination (square of correlation coefficient). **D**. Heat map depicting color-coded expression levels of two representative clusters of differentially expressed miRNAs between TAS cells (n=3) and PC cells, consisting of epithelial and stromal miRNA expression signatures. **E**. Quantitative real-time PCR validation of selected miRNAs in cultured PC cells (n=5) and in TAS cells (n=9).

miRNA expression patterns of pancreatic cancer cell lines have been previously described [19, 20], yet a comprehensive data set describing miRNA expression in TAS cells is lacking. We thus sought to determine the differences in miRNA expression between TAS cells and pancreatic cancer cells. miRNA profiles were established using nCounter miRNA assay containing 800 human miRNA probes. One hundred fifty-eight (approximately 20%) miRNAs were detected above threshold and considered expressed in the human PC cell line L3.6pl (Table 1). Similarly, an average of 149 miRNAs were detected in three TAS cell lines (range 128-158), of which one hundred sixteen miRNAs were present in all three TAS cell lines (Figure 1A). Supplemental Table 1 summarizes the top 100 miRNAs that were expressed in the TAS and PC cell lines.

**Table 1:** Establishment of miRnomes expression in PC cells and TAS cells by nCounter miRNA expression assay

	Cell Types						Co-cultured Cells									
	TAS Cells					PC cells	co-cultured TAS Cells (ESA- sorted)					co-cultured PC Cells (ESA+ sorted)				
	TAS1	TAS2	TAS3	Mean	SD	L3.6pl	TAS1	TAS2	TAS3	Mean	SD	TAS1	TAS2	TAS3	Mean	SD
Threshold value	70.57	111.31	98.92	93.60	20.88	90.12	70.40	78.46	100.97	84.99	15.84	72.57	116.67	84.37	91.20	29.43
miRNAs detected	157	139	150	149	9.07	158	158	152	154	156	3.06	158	128	128	138	52.64
% of detected miRNAs	19.6	17.4	18.8	18.6	1.13	19.8	19.8	19.0	19.3	19.4	0.38	19.8	16.0	16.0	17.3	6.58

We next performed a global comparison of the expression data between the two types of cells (Figure 1B and 1C). The presence of expression of 89 miRNAs were shared by both TAS cells and cancer cells, representing a 56˜64% overlap in miRNA expression. This indicates that nearly half of detected miRNAs were differentially expressed between the two types of cells. Depicted in Figure 1C, the poor correlation between TAS cells and cancer cells (*r*^2^=0.073˜0.119) supports a differential miRNA expression between the two groups. In contrast, the expression of the three TAS cell lines had excellent correlation (*r*^2^>0.98). These data demonstrate differential miRNA expression between pancreatic cancer cells and TAS cells grown in isolation.

### Identification of epithelial and stromal miRNA expression signatures

Knowing that cancer cells and TAS cells differentially express miRNAs, we set out to identify individual miRNAs that were differently expressed between PC and TAS cells (n = 3). The data set of 158 miRNAs expressed in cultured PC cells and the data set of 116 miRNAs expressed in all cultured TAS cells were compared using the nSolver program. The difference in mean ratio of nCounter digital readings was calculated for each miRNA, and all miRNAs were ranked according to the difference in expression. Table 2 lists the top 30 differentially expressed miRNAs between the two cell types. As illustrated in Figure 1C, miR-205 and miR-200 family members, particularly miR-200b/-200c, were highly expressed in PC cells but lack expression in TAS cells. Conversely, miR-145 and miR-199 family members (miR-199a/199b) were the most differentially expressed miRNAs in TAS cells as compared to PC cells. Unsupervised hierarchical clustering identified two miRNA clusters that were specific to PC and TAS cells, respectively (Figure 1D). The natural log difference in digital readings between the two (Table 2) suggests a strong association of the signature miRNAs with the epithelial or stromal phenotype. Quantitative RT-PCR validation, performed on a larger sampling of multiple PC and TAS cell lines (Figure 1E), confirmed the signature miRNA expressions in specific cell types.

**Table 2:** Top 30 most differentially expressed miRNAs identified between TAS cells and PC cells in this study

Top 15 highly expressed in TAS cells			Top 15 highly expressed in PC cells			
miRNA probe ID	Accession no.	Ratio	miRNA probe ID	Accession no.	Ratio
hsa-miR-145-5p	MIMAT0000437	8980.05	hsa-miR-205-5p	MIMAT0000266	23077.19
hsa-miR-199a-5p	MIMAT0000231	1563.68	hsa-miR-200c-3p	MIMAT0000617	5968.64
hsa-miR-199b-5p	MIMAT0000263	296.31	hsa-miR-200b-3p	MIMAT0000318	467.15
hsa-miR-136-5p	MIMAT0000448	228.24	hsa-miR-429	MIMAT0001536	171.07
hsa-miR-137	MIMAT0000429	227.70	hsa-miR-196b-5p	MIMAT0001080	116.42
hsa-miR-139-5p	MIMAT0000250	216.83	hsa-miR-582-5p	MIMAT0003247	99.53
hsa-miR-127-3p	MIMAT0000446	159.72	hsa-miR-135b-5p	MIMAT0000758	87.82
hsa-miR-337-5p	MIMAT0004695	148.57	hsa-miR-182-5p	MIMAT0000259	55.81
hsa-miR-139-3p	MIMAT0004552	146.36	hsa-miR-96-5p	MIMAT0000095	53.82
hsa-miR-487b	MIMAT0003180	118.72	hsa-miR-200a-3p	MIMAT0000682	44.88
hsa-miR-199a-3p+hsa-miR-199b-3p	MIMAT0000232	107.49	hsa-miR-4284	MIMAT0016915	41.90
hsa-miR-1233	MIMAT0005588	66.04	hsa-miR-345-5p	MIMAT0000772	27.99
hsa-miR-379-5p	MIMAT0000733	62.89	hsa-miR-378g	MIMAT0018937	25.01
hsa-miR-660-5p	MIMAT0003338	62.50	hsa-miR-203	MIMAT0000264	22.43
hsa-miR-335-5p	MIMAT0000765	60.53	hsa-miR-141-3p	MIMAT0000432	21.69

### Differential miRNA expression in human pancreatic cancer tissue specimens

**Figure 2:**
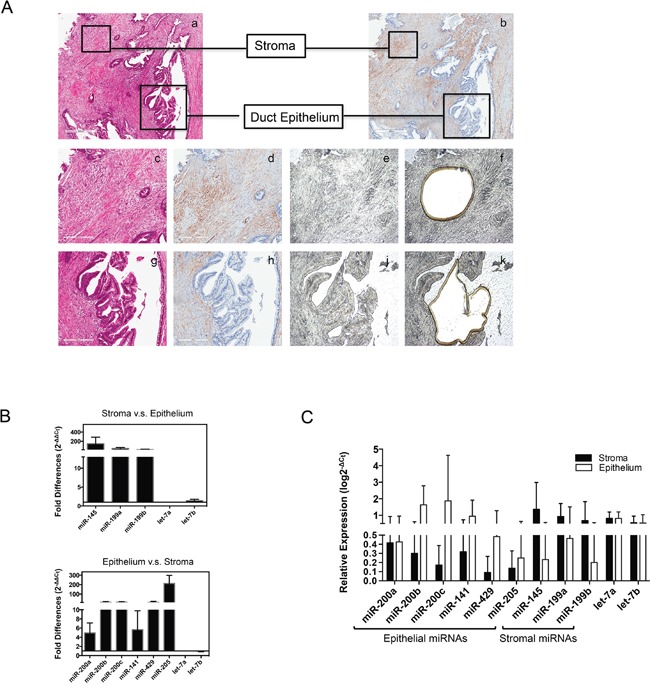
Validation of candidate epithelial and stromal miRNAs **A**. Laser capture microdissection for the separation of epithelial and stromal tissues from human pancreatic cancer. Image a & b (40x magnification) are hematoxylin/eosin staining and α-smooth muscle actin (α-SMA) staining; Image c, d, g and h (100x magnification) are enlarged for selected stromal and duct epithelial compartment staining with hematoxylin/eosin staining (c and g) and α-SMA staining (d and h); PDAC tissue section (10 μm) on PEM membrane before (image e and j) and after (image f and k) microdissection, unstained. **B**. and **C**. Quantitative real-time PCR determination of candidate miRNAs from LCM captured tissue sections, presented as comparative expression level (B) and relative expression level (C). All PCR tests were conducted in triplicate and repeated twice. Data presented are normalized to let-7a expression in each sample, shown in means with SD. Fold changes were calculated using ΔΔCt method. Relative expression level was calculated using ΔCt method related to let-7a expression.

Since the above data suggested that miR-205 and miR-200 family members are associated with epithelial cells, and miR-145 and miR-199 are associated with stromal cells, we reasoned that these miRNAs could potentially represent epithelial and stromal miRNA expression signatures. Each of these putative signature miRNAs has been specifically associated with pancreatic cancer [12, 19, 21]. We thus used quantitative real-time PCR to further explore the expression of miRNAs of interest *in situ*. To validate these cell-type specific miRNA expression patterns in surgical samples, epithelial and stromal compartments of PDAC were separated using laser capture micro-dissection (LCM). To minimize the potential for sampling contamination from isolated tumor cells, only well-differentiated pancreatic adenocarcinoma tissues (n=4) were selected for this study. Two of the patients were T3N1M0 and two were T3N0M0. Lymphovascular invasion was observed in all cases. Serial sections assisted in defining the pancreatic adenocarcinoma epithelial and α-SMA positive stromal regions for LCM as shown in Figure 2A. In line with *in vitro* data, qRT-PCR detected high expression of the miR-200 family members and miR-205 in epithelial tissue micro-dissections compared to stromal tissues (average fold difference of 5- to 140- higher in epithelium, *p* < 0.05), and the members of miR-199 family and miR-145 highly expressed in the stromal compartment in comparison with epithelium with a fold difference ranging from 21- to 241- higher (*p* < 0.05, Figure 2B). However, in contrast to the *in vitro* data (Figure 1E), we detected expression of low levels of PC epithelial signature miRNAs in stromal compartment, and vice versa; low expression levels of the candidate stromal signature miRNAs were also detected in the PC epithelium compartment (Figure 2C).

### Cell-cell interactions alter miRNA expression in co-cultured cancer cells and TAS cells

**Figure 3:**
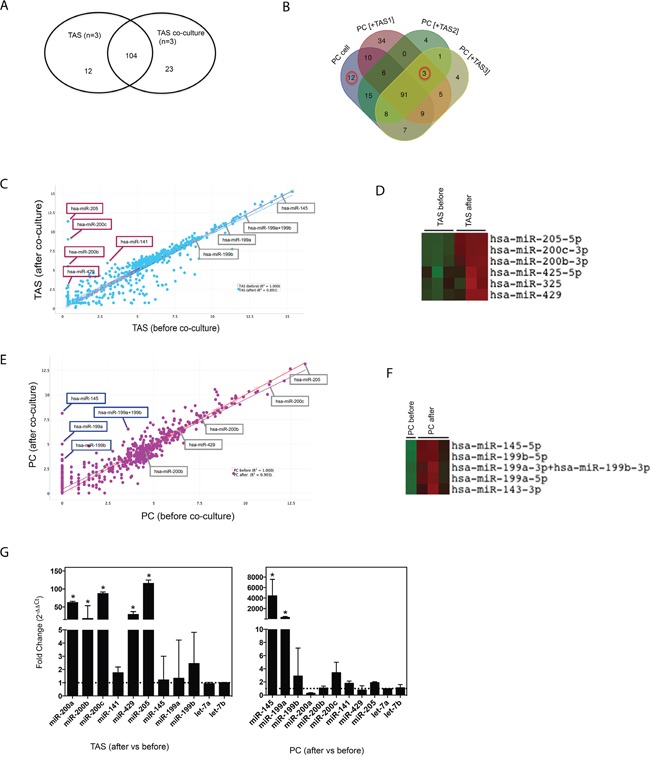
Co-culture alters miRNA concentrations Venn diagram comparison for miRNA expression in TAS **(A)** and in PC cells **(B)** before and after co-culture. Scatter plot analysis depicting miRNA expressions in TAS cell lines **(C)** and PC cells **(E)** before and after co-culture. All miRNAs of interest are indicated. Data are plotted as log2 digital signal readings. *r^2^*: coefficient of determination (square of correlation of coefficient). **(D)** & **(F)** Representative clusters of miRNAs (rows) on cells before and after co-culture (columns). **(G)** qRT-PCR confirmation of miRNAs of interest in TAS cells (n=6) and PC cells (n=4). Fold changes were calculated using ΔΔCt method comparing specific miRNA expression levels in cells after co-culture with levels in cells without co-culture. Bars show mean ± SD. * *p* < 0.05.

To investigate how TAS and PC cell interactions impact miRNA expression, we co-cultured human PC and TAS cells in a monolayer [22] for 48 hrs. After the PC and TAS cells were separated into pure populations by flow cytometric sorting [22], miRNA expression profiling from the co-cultured TAS and PC cells was conducted and established based on the cut-off shown in Table 1 right panel. Data analysis revealed 12 down-regulated and 23 up-regulated miRNAs in the co-cultured TAS cells compared to TAS cells alone; and 12 down-regulated and 3 up-regulated miRNAs in the PC cells after co-culture compared to PC cells alone (Figure 3A, 3B and Table 3). Correlation of determination (*r^2^*) suggested the majority of miRNA expression levels remained unchanged (*r^2^* =0.891 for TAS cells and *r^2^* =0.901 for PC cells), yet several miRNAs were notably up- or down-regulated during co-culture (Figure 3C and 3E). Unsupervised hierarchical clustering analysis identified and confirmed the clusters of the up-regulated miRNAs for both TAS cells and cancer cells (Figure 3D and 3F). Interestingly, the most robust up-regulated miRNAs in co-cultured TAS cells were the miR-205 and the miR-200 family members, in particular miR-200c, -200b and -429, these made up the candidate epithelial expression signature miRNA identified. Likewise, the most robust up-regulated miRNAs in co-cultured PC cells made up the identified candidate stromal expression signature miRNAs: miR-145 and the miR-199 family members. qPCR validated the significantly up-regulated miRNAs in PC and TAS cells observed during the co-culture process (Figure 3G).

**Table 3:** Changed miRNA expression in PC and TAS cells led by co-culture

Cell types	Regulation	Number of changes	miRNA probe ID
PC cells	Down-	12	hsa-miR-196b-5p, hsa-miR-376b, hsa-miR-4741, hsa-miR-149-5p, hsa-miR-1228-3p, hsa-miR-302a-3p, hsa-miR-329, hsa-miR-422a, hsa-miR-2054, hsa-miR-575, hsa-miR-499b-3p, hsa-miR-551a
	Up-	3	hsa-miR-199b-3p, hsa-miR-199a-3p+ hsa-miR-145-5p
TAS cells	Down-	12	hsa-miR-4455, hsa-miR-548ah-5p, hsa-miR-324-5p, hsa-miR-1290, hsa-miR-548aa, hsa-miR-337-5p, hsa-miR-136-5p, hsa-miR-590-5p, hsa-miR-514b-5p, hsa-miR-338-3p, hsa-miR-548ai, hsa-miR-1253
	Up-	23	hsa-miR-193a-3p, hsa-miR-376b, hsa-miR-342-3p, hsa-miR-30a-5p, hsa-miR-553, hsa-miR-503, hsa-miR-200c-3p, hsa-miR-1228-3p, hsa-miR-92a-3p, hsa-miR-151a-5p, hsa-miR-4488, hsa-miR-383, hsa-miR-205-5p, hsa-miR-484, hsa-miR-573, hsa-miR-330-5p, hsa-miR-200b-5p, hsa-miR-433, hsa-miR-662, hsa-miR-380-3p, hsa-miR-760, hsa-miR-583, hsa-miR-19a-3p

### Exogenous expression of miR-200b/-200c/-205 induces cytokine production by TAS cells

**Figure 4:**
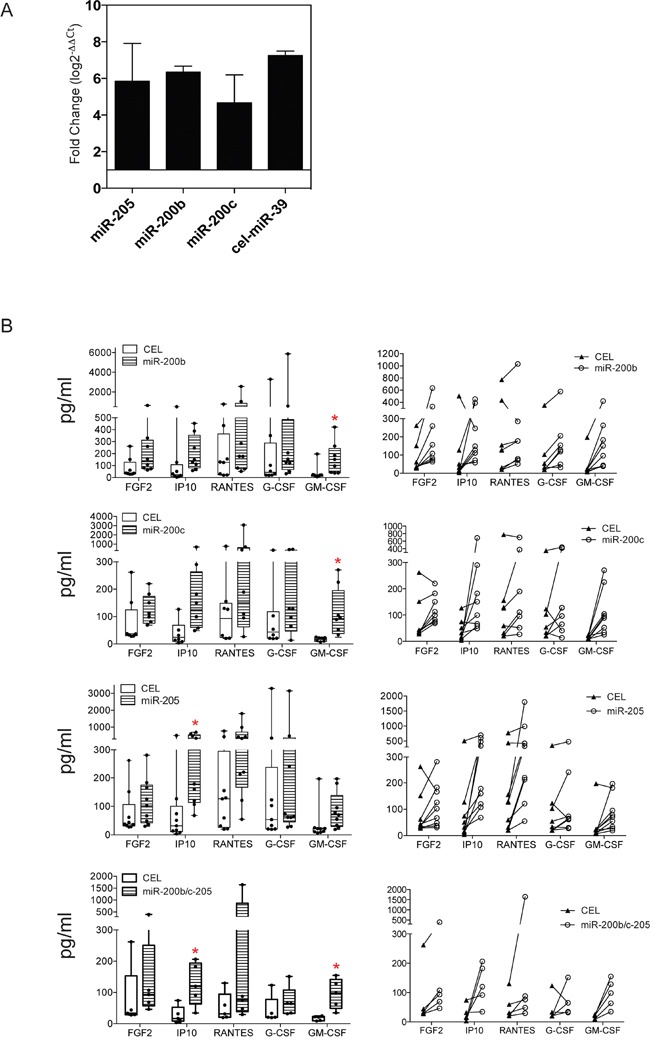
Ectopic expression of miR-200b/-200c and -205 induces cytokine production in TAS cells **A**. Confirmation of successful transfection of miRNAs miR-200b, miR-200c, miR-205, as well as scramble control of cel-miR-39 of TAS cells by quantitative real-time PCR. Data were normalized to let-7a and presented as means ± SD from triplicate of eight transfections. Fold changes are relative to non-transfected cells. **B**. Millipore Luminex multi-plex assay for quantitative measurements of cytokines in conditioned media of TAS cells transfected with cel-miR-39 (CEL) or miR-200b, -200c, -205 or the pool of the three miRNAs. Left panel shows mean ± SD of grouped data, and right panel plotted each individual sample paired with its control. Red * indicates *p* < 0.05.

In our previous work, we demonstrated that co-culture of cancer cells and TAS cells enhanced the secretion of certain cytokines by the TAS cells [22]. Thus, we asked whether this altered cytokine secretion in TAS cells following co-culture was the result, in part, of elevated miR-200b, miR-200c and miR-205 expression. qRT-PCR confirmed the successful over-expression by lipofectamine transfection of the specific miRNAs as well as mock control of cel-miR-39 (CEL) in TAS cells (Figure 4A). Using the Luminex 16-multiplex analysis, we observed increased production of five cytokines in all three miRNA transfected TAS cells. These were FGF2, IP10, RANTES, G-CSF, and GM-CSF (Figure 4B). However, statistically significant increased production was only observed for IP10 in miR-205 transfected TAS cells, and for GM-CSF in miR-200b or miR-200c transfected TAS cells (Table 4). The production of these five cytokines was also augmented in TAS cells transfected simultaneously with the pool of three miR-200b/-200c and miR-205 oligonucleotides (Figure 4B), yet again, only IP10 and GM-CSF were significantly increased (Table 4).

**Table 4:** Changed cytokines secretion caused by exogenous miRNAs transfection

		Significant	P value	Mean (CEL)	Mean (miR-)	Difference	SE of difference	t ratio
**miR-200b**	FGF2		0.10	80.03	213.65	-133.62	75.32	1.77
	IP10		0.21	94.66	200.82	-106.16	79.95	1.33
	RANTES		0.30	211.35	554.97	-343.62	322.46	1.07
	G-CSF		0.63	487.18	892.50	-405.31	819.97	0.49
	GM-CSF	*	0.05	39.75	154.57	-114.83	52.63	2.18
**miR-200c**	FGF2		0.22	77.82	123.54	-45.71	35.88	1.27
	IP10		0.06	40.78	197.84	-157.06	76.84	2.04
	RANTES		0.29	164.46	578.36	-413.90	376.24	1.10
	G-CSF		0.31	90.68	165.98	-75.29	71.08	1.06
	GM-CSF	*	0.01	16.20	112.06	-95.86	31.74	3.02
**miR-205**	FGF2		0.32	76.03	115.67	-39.64	38.40	1.03
	IP10	*	0.04	92.39	305.53	-213.14	94.51	2.26
	RANTES		0.14	194.47	508.62	-314.15	203.72	1.54
	G-CSF		0.97	446.72	465.18	-18.46	493.10	0.04
	GM-CSF		0.13	36.28	83.98	-47.69	29.57	1.61
**miR-200b/c+205**	FGF2		0.45	79.35	142.04	-62.69	78.77	0.80
	IP10	*	0.02	28.06	126.86	-98.80	33.37	2.96
	RANTES		0.34	52.41	377.48	-325.07	317.97	1.02
	G-CSF		0.40	43.50	69.52	-26.02	29.50	0.88
	GM-CSF	*	0.01	17.46	95.93	-78.47	21.84	3.59

### Exogenous expression of miR-200b/-200c/-205 inhibits TAS cell migration

**Figure 5:**
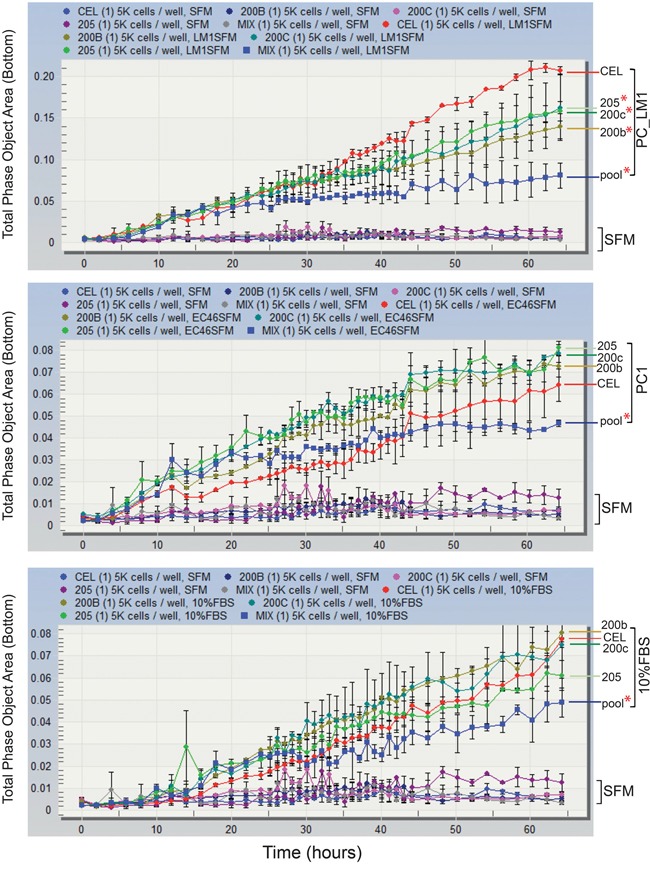
Pooled expression of miR-200b/-200c and -205 inhibits TAS cells migration TAS cells transfected with control (CEL) or individual specific miRNAs (miR-200b, -200c or -205) or the combination of the three (pool) were plated at a density of 5000 cells per well in the upper chamber of the ClearView cell migration plate. Serum-free conditioned medium from PC_LM1 (top panel), a cell line derived from a PDAC liver metastasis and PC1 (middle panel), a cell line derived from a primary neoplasm were added to the bottom reservoir. Serum-free (SFM) and 10% FBS supplemented medium (bottom panel) were served as negative and positive controls. Total phase cell-covered area was measured and plotted against incubation time (hours). Differences of migrated cells covered area between specific miRNA transfection to control miRNA (CEL) were compared and statistically analyzed. Red * indicates *p* < 0.05.

The miR-200 family is established in epithelial to mesenchymal transition (EMT) by direct targeting of *ZEB1* and *ZEB2* genes, and a component of this process is the inhibition of cell migration [23]. We thus sought to determine the effect of these miRNAs on TAS cell migration. Cell migration was induced with medium containing 10% FBS, as well as conditioned media from primary pancreatic cancer cell (PC_1) cultures and pancreatic cancer liver metastasis cell (PC_LM1) cultures, and measured using a live cell imaging system. As shown in Figure 5, serum-free medium (SFM) had no effect on cell migration. 10% FBS (Figure 5 bottom panel) and PC_1 conditioned media (Figure 5 middle panel) induced cell migration, and a significant reduction in migration was observed following pooled transfection with the three miRNAs (miR-200b/-200c/-205). Individual miRNA transfection did not inhibit migration in these groups. PC_LM1 condition media robustly facilitated cell migration resulting in two to three-fold more surface coverage on the bottom membrane at 64-hours stimulation. In contrast to PC-1 conditioned media, PC_LM1-induced migration was significantly retarded by transfection of the individual of miRNA oligoes (Figure 5 top panel). The strongest inhibition (2.6-fold reduction, *p* < 0.001) was found in the transfection of the pool of the three miRNAs.

## DISCUSSION

Dysregulated miRNA expression in PDAC suggests that miRNAs contribute to initiation and progression of pancreatic cancer. Translational applications of these findings include seeking diagnostic and/or prognostic biomarkers, as well as therapeutic targets [21, 24]. To date, these studies have either used cultured cancer cells or whole tissue specimens that contained both epithelial and stromal elements. We sought to identify differences in miRNA expression between pancreatic cancer cells and the associated stroma in order to gain a better understanding of roles of miRNAs in the complex heterogeneous pancreatic tumor microenvironment. Our data demonstrated a distinct epithelial miRNA expression pattern in PC cells that differentiate them from primary cultured TAS cells. We further identified two miRNA clusters of miR-200 family/miR-205 and miR-199 family/miR-145 which demonstrated significant discrimination between the two cell types. These findings were further validated using quantitative real-time PCR and extended to human pancreatic cancer tissue specimens. The observed expression levels of miR-200 and miR-205 in ductal epithelium, and vice versa, miR-145 and miR-199 expression in stroma further support the notion of cell type-specific miRNA signatures.

In line with these data, the miR-200 family is documented as an epithelial marker in other solid tumors [25], and their function of suppressing epithelial-to-mesenchymal transition (EMT) is broadly accepted [26]. The miR-200 family can be functionally grouped into two subfamilies according to the presence of the specific seed sequence: miR-200a/-141, and miR-200b/-200c/-429 [25]. Our data show that in samples of pancreatic cancer cells, the miR-200b/-200c/-429 cluster has a stronger association than the miR-200a/-141 subfamily. miR-205 is also regarded as a specific marker of epithelial malignancy, particularly in squamous cancer of the oropharnyx [27]. There is a relative lack of miRNA markers for stroma, but miR-145 and miR-199 have been reported to be confined to human mesenchymal stromal cells and fibroblasts [28, 29], with putative roles in regulating metastasis-associated genes such as mucin 1 and B16F10 [30, 31]. All these miRNAs were previously reported as over-expressed in pancreatic cancer and chronic pancreatitis [12].

Central to this work is an overall lack of understanding of how interactions between cells in the tumor microenvironment impact their biology, and the role of miRNAs in this regulation is only beginning to be understood [32]. Mitra and colleagues provided solid evidence of cancer cells altering fibroblast biology through the action of miRNAs in the ovarian cancer microenvironment [33]. Thus, we explored the impact of changes in miRNA expression following co-culture. The identification of specific miRNAs in cells following co-culture, which were previously absent in PC or TAS cells from pure cultures, suggests the possibility of an exchange of miRNAs between neighboring cells. This finding was supported by laser capture micro-dissection of surgical specimens that detected low levels of these miRNAs in neighboring compartments. Taken together, this observation supports a potential mechanism of microRNAs acting as paracrine signaling molecules in cell-cell communication in the pancreatic cancer microenvironment.

If miRNAs act as paracrine messages, a potential sequela in TAS would be alterations in secreted protein production. We have previously reported that the co-culture model used in this work results in significant changes in the production of several cytokines [22, 34]. Here we demonstrate that the production and release of five cytokines were increased in TAS cells following induced expression of miR-200b, miR-200c and miR-205. These data also suggest a potential synergistic function between miR-200 and miR-205. The synergistic effect of the three miRNAs also appeared in cell migration experiments, where a pooled transfection of three miRNAs inhibited migration beyond that of individual miRNA transfection. This synergy has been previously proposed in pancreatic cancer and other tumors, and associates with worsened prognosis [35–37]. In our model, this led to a statically significant augmentation of cytokines GM-CSF and IP10 following a pooled transfection of miR-200b/-200c and miR-205. These data implicate that miRNA regulation may alter the cytokine milieu within the tumor microenvironment.

There are a number of limitations that must be considered when drawing conclusions from this work. First, the source of TAS cultures is neoplastic and the cells are intrinsically activated, thus miRNA expression patterns identified should not be considered to represent quiescent or benign expression patterns. Second, in co-culture experiments, the lack of a reliable surface marker for TAS cells forced the use of negative selection, thus risking potential contamination with cancer cells expressing low levels of ESA. However, we have previously reported a significant difference in ESA positivity by cytometric analysis that facilitates this approach with minimal risk of ESA^dim^ PC cells contaminating the sample [22]. Third, there is a current lack of a pre-clinical *in vivo* murine model to validate our findings. While patient-derived xenografts maintain an architecture that includes a robust stroma, the stromal elements are of murine origin [38] and we lack a mechanism to stably induce miR-200b/c or miR205 expression *in vivo*. Finally, while our data hint that an exchange of miRNA between cells may be occurring, the present methodology does not exclude changes in transcription in response to protein or other paracrine signals. If this does represent a transfer of miRNAs between cells, an exosome-mediated mechanism needs to be considered. Considerable work remains to elucidate the mechanism(s) of miRNA expression level changes in response to co-culture.

## MATERIALS AND METHODS

### Cell culture and monolayer co-culture

Primary human pancreatic tumor-associated stromal cells were isolated using outgrowth methods and cultured as previously described [22]. Only early passage (passage 2-3) stromal cells (TAS) were used in this study. Human immortal pancreatic cancer (PC) cell lines (L3.6pl and BxPC3), and primary xenograft-isolated cancer cell lines from patients with primary tumor (PC1) and liver metastasis pancreatic cancer cell lines (PC_LM1 and PC_LM2) were cultured in DMEM-F12 supplemented with 10% FBS. For the monolayer co-culture, PC cancer cell lines and TAS cells were deposited on the culture surface at a ratio of 1:10 for immortal PC cell lines and 1:5 for xenograft cell lines respectively.

### Fluorescence-activated cell sorting (FACS)

In order to separate co-culture conditioned PC cells and TAS cells, all cells were detached from the culture surface with 0.05% trypsin/0.01% EDTA solution and then labeled with PE conjugated anti-ESA (epithelial surface antigen) antibody. FACS was performed as described previously [22]. ESA^+^ (representing PC cells) and ESA^-^ (representing TAS cells) were collected separately. Cells were washed in PBS twice and subjected to RNA extraction.

### Laser capture microdissection

Well-differentiatedPDAC tissue specimens (n=4) were obtained by surgical resection using an IRB-approved protocol. Embedded, frozen tissue specimens were cut as a series of 10 μm thick sections and immediately fixed in 100% methanol. Microdissections were conducted by a pathologist who specializes in pancreatic diseases. To ensure accurate microdissection, two serial sections stained with hematoxylin/eosin or with α-SMA (α-smooth muscle actin) were used for guidance. Areas containing malignant ductal epithelial cells versus the stromal compartment (total area of 1.5-2×10^6^ um^2^ each) were independently dissected using the Leica Laser Microdissection system LMD6500 (Buffalo Grove, IL).

### Total cellular RNA (including miRNA) extraction

Cultured cells and LCM dissected samples were homogenized in Trizol (Invitrogen, Carlsbad, California) followed by phenol-chloroform phase separation. The integrity of the extracted total RNA was determined with the Agilent RNA 6000 Nano assay. miRNA concentration and miRNA/small RNA ratio was determined with the small RNA assay, both performed on Agilent 2100 Bioanalyzer (Agilent Technologies, Waldbronn, Germany).

### miRNA expression profiling

Extracted RNAs that passed a quality metric (RIN ≥ 7) were subjected to analysis following the manufacturer's protocols. Comprehensive miRNA expression data were collected using nCounter miRNA Expression Assay (HSA miRNA v2 Assay kit, NanoString, Seattle, WA) for the detection of 800 individual human miRNA from miRBase v.18. The code set also contains assay controls including house-keeping genes, positive and negative controls and non-human microRNAs. Concentrations of expressed miRNA in each sample were depicted by digital counts generated by the nCounter Digital Analyzer, and normalized by top 100 expression analysis. The detection threshold for each sample was calculated as three standard deviations of the mean count of the negative controls, and a miRNA was deemed expressed if its normalized counter was above the detection threshold.

### Quantitative real-time PCR validation

Candidate, differentially expressedmiRNAs were individually validated using quantitative real-time PCR (qRT-PCR) for the validation of nCounter analysis. Reverse transcription was performed from the RNA samples using Universal cDNA synthesis kit (Exiqon, Denmark) according to the manufacturer's instructions. miRNAs were amplified with predesigned primer sets (Supplemental Table 2) and miRCURY LNA™ Universal RT microRNA PCR system (Exiqon) following manufacturer's instruction, and amplifications were carried out on a Mx3005p thermocycler (Strategene, La Jolla, CA). Each sample was run in triplicates and repeated twice. Relative expression of miRNAs was analyzed using the comparative C_T_ method and was normalized to hsa-let-7a, a miRNA that is consistently expressed in both stromal and cancer cells. Fold differences between groups were calculated using ΔΔC_T_methods.

### miRNA transfection

HumanTAS cells were plated in 6-well plate at density of 2.5 × 10^5^ cells per well and incubated overnight. Cells were transfected with synthetic miRNA mimics (Exiqon) miR-200b, miR-200c, miR-205, or a pool of the three at a final concentration of 25 pmol per well using RNAiMAX transfection reagent (Invitrogen). Synthetic miRNA mimic from *C. Elegans* (Cel-miR-39) served as a control. RNAs were extracted from remaining cells to assess the miRNA mimics transfection.

### Analysis of secreted cytokines

Serum-free medium (0.5 ml) was incubated with the miRNA mimics transfected cells for 24-hours, and the supernatants were collected for measurement of cytokine secretion. Based on our previous observation of the effects of co-culture upon cytokine secretion [22], a 16-plex immuno-multiplex assay was custom designed and purchased from Millipore (Billerica, MA), including FGF-2, Eotaxin (CCL11), G-CSF, GM-CSF, IP10 (CXCL10), MIP1α (CCL3), RANTES (CCL5), VEGF, IFNα2, IL-10, MDC (CCL22), PDGF-AA, PDGF-AB/BB, sCD40L, IL-1RA, and IL-7. Cyotokine concentrations (pg/ml) were determined using BeadView™ software (Millipore).

### Chemotactic migration assay

Quantification of cell migration was performed using the IncuCyte™ chemotaxis Assay (Essen Bioscience, MI) following manufacturer's instructions. In brief, TAS cells transfected with specific or control miRNAs mimics were trypsinized after 36-hours of transfection and seeded into the 96-well cell migration plate (IncuCyte™ ClearView, Essen Bioscience, MI) with 5000 cells/well on PET membrane on top chamber. Conditioned media of from PC_LM1 and PC1 cell cultures were added to the reservoir plate with serum-free medium and 10% FBS as assay control. Whole-well images of cells on the bottom of the ClearView plate™ membrane were captured every 2-hours until 64-hours using IncuCyte ZOOM™ instrument.

### Statistical analysis

Comprehensive miRNA expression profiling was generated and compared between different groups using nSolve software (nanoString Technologies, Seattle, WA). Hierarchical clustering was performed using Pearson's correlation. All statistical analysis was conducted using Prism v6 software. Statistically significance was defined as a probability of *p* < 0.05.

This work utilized a LEICA 7000 laser microdissection microscope that was purchased with funding from a National Institutes of Health SIG grant 1S10OD016350-01.

## SUPPLEMENTARY MATERIALS TABLES




