# Supplementary material for: The pancreatic tumor microenvironment drives changes in miRNA expression that promote cytokine production and inhibit migration by the tumor associated stroma
Source: Oncotarget. 2016 Jul 20;8(33):54054–67. doi: 10.18632/oncotarget.10722 (PMC5589562; doi:10.18632/oncotarget.10722)
Supplement: Supplementary file 1 [file oncotarget-08-54054-s001.pdf]

The pancreatic tumor microenvironment drives changes in miRNA expression that promote cytokine production and inhibit migration by the tumor associated stroma

SUPPLEMENTARY TABLES

Supplementary Table S1: Top 100 Expressed miRNAs for TAS Cell Lines and PC Cell Line L3.6pl

See Supplementary File 1

Supplementary Table 2: Information of Primers for real-time PCR

Primer ID	Target sequnces	*Product no.	Batch no.
hsa-miR-145-5p	GUCCAGUUUUCCCAGGAAUCCCU	204483	230863
hsa-miR-199a-5p	CCCAGUGUUCAGACUACCUGUUC	204494	225157
hsa-miR-199b-5p	CCCAGUGUUUAGACUAUCUGUUC	204152	232352
hsa-miR-200a-3p	UAACACUGUCUGGUAACGAUGU	204707	222848
hsa-miR-200b-3p	UAAUACUGCCUGGUAAUGAUGA	206071	227873
hsa-miR-200c-3p	UAAUACUGCCGGGUAAUGAUGGA	204482	229141
hsa-miR-141-3p	UAACACUGUCUGGUAAAGAUGG	204504	222854
hsa-miR-429	UAAUACUGUCUGGUAAAACCGU	205901	225550
hsa-miR-205-5p	UCCUUCAUUCCACCGGAGUCUG	204487	225471
hsa-let-7a-5p	UGAGGUAGUAGGUUGUAUAGUU	205727	228552
hsa-let-7b-5p	UGAGGUAGUAGGUUGUGUGGUU	204750	228235
cel- miR-39-3p	UCACCGGGUGUAAAUCAGCUUG	203952	229853

* miRCURY LNA™ Universal RT microRNA PCR LNA™ PCR primers set (Exiqon, Foster City, CA)
