# Supplementary material for: The pancreatic tumor microenvironment drives changes in miRNA expression that promote cytokine production and inhibit migration by the tumor associated stroma
Source: Oncotarget. 2016 Jul 20;8(33):54054–67. doi: 10.18632/oncotarget.10722 (PMC5589562; doi:10.18632/oncotarget.10722)
Supplement: Supplementary file 2 [file oncotarget-08-54054-s002.docx]

Supplementary Table1. Top 100 Expressed miRNAs for TAS Cell Lines and PC Cell Line L3.6pl

| **TAS Cells** | | | | | | |  | **PC Cells** | | |
| --- | --- | --- | --- | --- | --- | --- | --- | --- | --- | --- |
| **miRNA probe ID** | **Accession no.** | **TAS1** | **TAS2** | **TAS3** | **Mean** | **SD** |  | **miRNA probe ID** | **Accession no.** | **L3.6pl** |
| hsa-miR-125b-5p | MIMAT0000423 | 37090.1 | 38450.7 | 31263.1 | 35601.3 | 3818.1 |  | hsa-miR-205-5p | MIMAT0000266 | 24538.8 |
| hsa-let-7a-5p | MIMAT0000062 | 18090.0 | 21974.6 | 25352.7 | 21805.8 | 3634.3 |  | hsa-let-7a-5p | MIMAT0000062 | 14064.5 |
| hsa-miR-21-5p | MIMAT0000076 | 14512.8 | 11748.9 | 24318.1 | 16859.9 | 6605.1 |  | hsa-miR-4454 | MIMAT0018976 | 11125.3 |
| hsa-miR-100-5p | MIMAT0000098 | 12634.1 | 12907.7 | 14670.6 | 13404.2 | 1105.3 |  | hsa-let-7b-5p | MIMAT0000063 | 6515.7 |
| hsa-miR-145-5p | MIMAT0000437 | 4631.6 | 12318.5 | 11606.4 | 9518.9 | 4247.4 |  | hsa-miR-200c-3p | MIMAT0000617 | 6346.7 |
| hsa-let-7b-5p | MIMAT0000063 | 6032.0 | 9514.0 | 9328.3 | 8291.4 | 1958.9 |  | hsa-miR-720 | MIMAT0005954 | 5885.0 |
| hsa-miR-4454 | MIMAT0018976 | 4944.2 | 6526.2 | 7715.5 | 6395.3 | 1390.3 |  | hsa-miR-15b-5p | MIMAT0000417 | 4059.3 |
| hsa-miR-143-3p | MIMAT0000435 | 2098.1 | 5556.3 | 5642.0 | 4432.1 | 2021.7 |  | hsa-miR-106a-5p+17-5p | MIMAT0000103 | 3807.9 |
| hsa-miR-29b-3p | MIMAT0000100 | 2526.4 | 4434.6 | 6061.2 | 4340.7 | 1769.3 |  | hsa-miR-20a-5p+20b-5p | MIMAT0000075 | 2925.7 |
| hsa-miR-23a-3p | MIMAT0000078 | 3119.2 | 2526.5 | 4128.0 | 3257.9 | 809.7 |  | hsa-let-7g-5p | MIMAT0000414 | 2365.7 |
| hsa-miR-199a-3p+199b-3p | MIMAT0000232 | 2993.9 | 3589.5 | 2671.6 | 3085.0 | 465.7 |  | hsa-miR-93-5p | MIMAT0000093 | 2091.0 |
| hsa-let-7g-5p | MIMAT0000414 | 2190.0 | 2813.2 | 3374.0 | 2792.4 | 592.3 |  | hsa-miR-29b-3p | MIMAT0000100 | 1813.2 |
| hsa-miR-378e | MIMAT0018927 | 6547.3 | 773.8 | 696.1 | 2672.4 | 3356.0 |  | hsa-miR-16-5p | MIMAT0000069 | 1796.3 |
| hsa-miR-720 | MIMAT0005954 | 2199.5 | 3012.4 | 2535.5 | 2582.5 | 408.5 |  | hsa-miR-19b-3p | MIMAT0000074 | 1781.5 |
| hsa-miR-125a-5p | MIMAT0000443 | 1457.6 | 3198.5 | 2729.1 | 2461.7 | 900.7 |  | hsa-miR-374a-5p | MIMAT0000727 | 1706.4 |
| hsa-miR-548ai | MIMAT0018989 | 1794.0 | 4072.6 | 1498.3 | 2454.9 | 1408.7 |  | hsa-miR-23a-3p | MIMAT0000078 | 1549.0 |
| hsa-let-7i-5p | MIMAT0000415 | 2376.1 | 2805.7 | 2133.1 | 2438.3 | 340.6 |  | hsa-miR-548ai | MIMAT0018989 | 1544.8 |
| hsa-miR-221-3p | MIMAT0000278 | 1634.1 | 3121.3 | 2187.2 | 2314.2 | 751.7 |  | hsa-miR-24-3p | MIMAT0000080 | 1263.8 |
| hsa-miR-22-3p | MIMAT0000077 | 1916.8 | 2151.4 | 2602.3 | 2223.5 | 348.4 |  | hsa-miR-125a-5p | MIMAT0000443 | 1217.3 |
| hsa-miR-199a-5p | MIMAT0000231 | 1275.1 | 2008.1 | 1689.3 | 1657.5 | 367.5 |  | hsa-miR-130a-3p | MIMAT0000425 | 1087.3 |
| hsa-miR-27b-3p | MIMAT0000419 | 1095.0 | 1725.1 | 2066.3 | 1628.8 | 492.8 |  | hsa-miR-100-5p | MIMAT0000098 | 1038.7 |
| hsa-miR-29a-3p | MIMAT0000086 | 1299.0 | 2142.1 | 1317.4 | 1586.1 | 481.5 |  | hsa-miR-191-5p | MIMAT0000440 | 1023.9 |
| hsa-miR-26a-5p | MIMAT0000082 | 1337.1 | 1461.7 | 1703.7 | 1500.8 | 186.4 |  | hsa-miR-221-3p | MIMAT0000278 | 933.1 |
| hsa-let-7e-5p | MIMAT0000066 | 948.3 | 1495.2 | 1597.2 | 1346.9 | 349.0 |  | hsa-let-7f-5p | MIMAT0000067 | 928.9 |
| hsa-miR-1283 | MIMAT0005799 | 3220.6 | 112.9 | 271.8 | 1201.8 | 1750.1 |  | hsa-miR-125b-5p | MIMAT0000423 | 927.8 |
| hsa-miR-24-3p | MIMAT0000080 | 885.1 | 1292.3 | 1411.2 | 1196.2 | 275.9 |  | hsa-miR-106b-5p | MIMAT0000680 | 923.6 |
| hsa-miR-130a-3p | MIMAT0000425 | 553.5 | 1165.7 | 1494.9 | 1071.4 | 477.8 |  | hsa-miR-15a-5p | MIMAT0000068 | 895.0 |
| hsa-miR-374a-5p | MIMAT0000727 | 747.9 | 934.8 | 1500.0 | 1060.9 | 391.6 |  | hsa-miR-338-3p | MIMAT0000763 | 856.0 |
| hsa-miR-181a-5p | MIMAT0000256 | 1211.9 | 588.6 | 1325.9 | 1042.1 | 396.9 |  | hsa-miR-21-5p | MIMAT0000076 | 843.3 |
| hsa-miR-15b-5p | MIMAT0000417 | 657.2 | 1299.7 | 1147.5 | 1034.8 | 335.7 |  | hsa-miR-378e | MIMAT0018927 | 839.1 |
| hsa-miR-34a-5p | MIMAT0000255 | 650.1 | 1185.2 | 1008.9 | 948.1 | 272.7 |  | hsa-miR-25-3p | MIMAT0000081 | 826.4 |
| hsa-let-7f-5p | MIMAT0000067 | 706.1 | 1013.0 | 938.7 | 886.0 | 160.1 |  | hsa-miR-548aa | MIMAT0018447 | 822.1 |

Supplementary Table1. Top 100 Expressed miRNAs for TAS Cell Lines and PC Cell Line L3.6pl (continue 1)

| **TAS Cells** | | | | | | |  | **PC Cells** | | |
| --- | --- | --- | --- | --- | --- | --- | --- | --- | --- | --- |
| **miRNA probe ID** | **Accession no.** | **TAS1** | **TAS2** | **TAS3** | **Mean** | **SD** |  | **miRNA probe ID** | **Accession no.** | **L3.6pl** |
| hsa-miR-191-5p | MIMAT0000440 | 987.6 | 788.7 | 868.6 | 881.6 | 100.1 |  | hsa-let-7d-5p | MIMAT0000065 | 787.3 |
| hsa-miR-16-5p | MIMAT0000069 | 635.8 | 1087.5 | 897.3 | 873.5 | 226.8 |  | hsa-miR-92a-3p | MIMAT0000092 | 759.8 |
| hsa-miR-23b-3p | MIMAT0000418 | 854.0 | 471.3 | 793.3 | 706.2 | 205.7 |  | hsa-miR-203 | MIMAT0000264 | 601.3 |
| hsa-miR-376c | MIMAT0000720 | 708.5 | 677.9 | 669.1 | 685.2 | 20.7 |  | hsa-let-7e-5p | MIMAT0000066 | 597.1 |
| hsa-miR-1253 | MIMAT0005904 | 1730.8 | 201.4 | 102.7 | 678.3 | 912.8 |  | hsa-let-7i-5p | MIMAT0000415 | 532.7 |
| hsa-miR-2116-5p | MIMAT0011160 | 1510.1 | 117.6 | 55.4 | 561.0 | 822.5 |  | hsa-miR-1290 | MIMAT0005880 | 524.2 |
| hsa-miR-99b-5p | MIMAT0000689 | 446.1 | 599.8 | 630.2 | 558.7 | 98.7 |  | hsa-miR-99b-5p | MIMAT0000689 | 516.8 |
| hsa-miR-548aa | MIMAT0018447 | 650.1 | 691.0 | 227.0 | 522.7 | 256.9 |  | hsa-miR-200b-3p | MIMAT0000318 | 496.7 |
| hsa-miR-376a-3p | MIMAT0000729 | 518.9 | 415.5 | 565.1 | 499.8 | 76.6 |  | hsa-miR-30b-5p | MIMAT0000420 | 493.6 |
| hsa-miR-361-5p | MIMAT0000703 | 435.4 | 438.7 | 573.5 | 482.5 | 78.8 |  | hsa-miR-22-3p | MIMAT0000077 | 484.1 |
| hsa-miR-10a-5p | MIMAT0000253 | 262.4 | 675.1 | 502.5 | 480.0 | 207.3 |  | hsa-miR-29a-3p | MIMAT0000086 | 466.1 |
| hsa-miR-1290 | MIMAT0005880 | 832.6 | 394.0 | 184.7 | 470.4 | 330.6 |  | hsa-miR-19a-3p | MIMAT0000073 | 458.7 |
| hsa-miR-1260a | MIMAT0005911 | 499.8 | 487.1 | 423.1 | 470.0 | 41.1 |  | hsa-miR-27b-3p | MIMAT0000419 | 427.0 |
| hsa-miR-550b-3p | MIMAT0018445 | 1197.6 | 110.1 | 100.2 | 469.3 | 630.7 |  | hsa-miR-18a-5p | MIMAT0000072 | 420.7 |
| hsa-miR-365a-3p | MIMAT0000710 | 325.6 | 616.5 | 462.8 | 468.3 | 145.5 |  | hsa-miR-376c | MIMAT0000720 | 416.4 |
| hsa-miR-214-3p | MIMAT0000271 | 399.6 | 543.9 | 363.1 | 435.5 | 95.6 |  | hsa-miR-26a-5p | MIMAT0000082 | 399.5 |
| hsa-miR-450a-5p | MIMAT0001545 | 226.6 | 547.6 | 515.2 | 429.8 | 176.7 |  | hsa-miR-148a-3p | MIMAT0000243 | 394.3 |
| hsa-miR-106a-5p+17-5p | MIMAT0000103 | 225.4 | 559.7 | 451.8 | 412.3 | 170.6 |  | hsa-miR-143-3p | MIMAT0000435 | 393.2 |
| hsa-miR-222-3p | MIMAT0000279 | 498.6 | 222.8 | 447.6 | 389.7 | 146.8 |  | hsa-miR-423-5p | MIMAT0004748 | 378.4 |
| hsa-miR-19b-3p | MIMAT0000074 | 188.5 | 517.8 | 418.0 | 374.8 | 168.9 |  | hsa-miR-107 | MIMAT0000104 | 377.4 |
| hsa-miR-132-3p | MIMAT0000426 | 411.5 | 292.6 | 399.4 | 367.8 | 65.5 |  | hsa-miR-374b-5p | MIMAT0004955 | 363.6 |
| hsa-miR-30b-5p | MIMAT0000420 | 287.5 | 314.0 | 420.5 | 340.7 | 70.4 |  | hsa-miR-148b-3p | MIMAT0000759 | 357.3 |
| hsa-miR-570-3p | MIMAT0003235 | 792.0 | 127.8 | 80.7 | 333.5 | 397.8 |  | hsa-miR-1260a | MIMAT0005911 | 335.1 |
| hsa-miR-148b-3p | MIMAT0000759 | 388.9 | 310.3 | 277.7 | 325.6 | 57.1 |  | hsa-miR-361-5p | MIMAT0000703 | 315.0 |
| hsa-miR-31-5p | MIMAT0000089 | 285.1 | 346.6 | 336.0 | 322.6 | 32.9 |  | hsa-miR-135b-5p | MIMAT0000758 | 280.2 |
| hsa-miR-409-3p | MIMAT0001639 | 207.6 | 298.2 | 452.7 | 319.5 | 123.9 |  | hsa-miR-519b-5p+519c-5p | MIMAT0005454 | 279.1 |
| hsa-miR-199b-5p | MIMAT0000263 | 223.1 | 377.3 | 341.9 | 314.1 | 80.8 |  | hsa-miR-1253 | MIMAT0005904 | 274.9 |
| hsa-miR-20a-5p+20b-5p | MIMAT0000075 | 169.4 | 447.1 | 320.8 | 312.4 | 139.0 |  | hsa-miR-98 | MIMAT0000096 | 267.5 |
| hsa-miR-98 | MIMAT0000096 | 330.4 | 266.5 | 338.5 | 311.8 | 39.4 |  | hsa-miR-4488 | MIMAT0019022 | 247.4 |
| hsa-miR-140-5p | MIMAT0000431 | 217.1 | 258.1 | 439.1 | 304.8 | 118.1 |  | hsa-miR-590-5p | MIMAT0003258 | 240.0 |
| hsa-miR-338-3p | MIMAT0000763 | 242.1 | 472.2 | 199.1 | 304.5 | 146.9 |  | hsa-miR-141-3p | MIMAT0000432 | 236.8 |
| hsa-let-7c | MIMAT0000064 | 289.9 | 341.0 | 281.9 | 304.2 | 32.1 |  | hsa-miR-331-3p | MIMAT0000760 | 236.8 |

Supplementary Table1. Top 100 Expressed miRNAs for TAS Cell Lines and PC Cell Line L3.6pl (continue 2)

| **TAS Cells** | | | | | | |  | **PC Cells** | | |
| --- | --- | --- | --- | --- | --- | --- | --- | --- | --- | --- |
| **miRNA probe ID** | **Accession no.** | **TAS1** | **TAS2** | **TAS3** | **Mean** | **SD** |  | **miRNA probe ID** | **Accession no.** | **L3.6pl** |
| hsa-miR-574-3p | MIMAT0003239 | 223.1 | 281.4 | 391.8 | 298.8 | 85.7 |  | hsa-miR-31-5p | MIMAT0000089 | 232.6 |
| hsa-let-7d-5p | MIMAT0000065 | 190.9 | 355.9 | 323.3 | 290.0 | 87.4 |  | hsa-miR-196a-5p | MIMAT0000226 | 226.3 |
| hsa-miR-503 | MIMAT0002874 | 23.9 | 625.8 | 194.8 | 281.5 | 310.2 |  | hsa-miR-23b-3p | MIMAT0000418 | 226.3 |
| hsa-miR-1246 | MIMAT0005898 | 755.0 | 9.6 | 71.4 | 278.7 | 413.7 |  | hsa-miR-4455 | MIMAT0018977 | 225.2 |
| hsa-miR-15a-5p | MIMAT0000068 | 134.8 | 308.4 | 370.7 | 271.3 | 122.2 |  | hsa-miR-365a-3p | MIMAT0000710 | 224.2 |
| hsa-miR-382-5p | MIMAT0000737 | 206.4 | 271.2 | 315.7 | 264.4 | 55.0 |  | hsa-miR-483-3p | MIMAT0002173 | 224.2 |
| hsa-miR-29c-3p | MIMAT0000681 | 301.8 | 220.9 | 248.1 | 256.9 | 41.2 |  | hsa-miR-888-5p | MIMAT0004916 | 222.0 |
| hsa-miR-93-5p | MIMAT0000093 | 149.1 | 301.9 | 309.8 | 253.6 | 90.6 |  | hsa-miR-511 | MIMAT0002808 | 212.5 |
| hsa-miR-302d-3p | MIMAT0000718 | 506.9 | 125.0 | 123.0 | 251.7 | 221.1 |  | hsa-let-7c | MIMAT0000064 | 211.5 |
| hsa-miR-337-3p | MIMAT0000754 | 268.4 | 217.2 | 262.5 | 249.3 | 28.0 |  | hsa-miR-1180 | MIMAT0005825 | 208.3 |
| hsa-miR-107 | MIMAT0000104 | 200.4 | 295.4 | 250.6 | 248.8 | 47.5 |  | hsa-miR-197-3p | MIMAT0000227 | 206.2 |
| hsa-miR-137 | MIMAT0000429 | 207.6 | 250.7 | 265.9 | 241.4 | 30.2 |  | hsa-miR-942 | MIMAT0004985 | 205.1 |
| hsa-miR-374b-5p | MIMAT0004955 | 176.5 | 203.2 | 314.9 | 231.5 | 73.4 |  | hsa-miR-151a-3p | MIMAT0000757 | 203.0 |
| hsa-miR-139-5p | MIMAT0000250 | 94.2 | 307.5 | 287.8 | 229.8 | 117.9 |  | hsa-miR-210 | MIMAT0000267 | 195.6 |
| hsa-miR-630 | MIMAT0003299 | 109.7 | 233.9 | 345.3 | 229.7 | 117.8 |  | hsa-miR-570-3p | MIMAT0003235 | 192.5 |
| hsa-miR-136-5p | MIMAT0000448 | 84.7 | 156.7 | 443.4 | 228.2 | 189.7 |  | hsa-miR-455-3p | MIMAT0004784 | 191.4 |
| hsa-miR-519b-5p+519c-5p | MIMAT0005454 | 157.5 | 310.3 | 183.9 | 217.2 | 81.7 |  | hsa-miR-520h | MIMAT0002867 | 190.4 |
| hsa-miR-25-3p | MIMAT0000081 | 202.8 | 209.7 | 231.2 | 214.6 | 14.8 |  | hsa-miR-429 | MIMAT0001536 | 181.9 |
| hsa-miR-888-5p | MIMAT0004916 | 442.5 | 139.0 | 60.4 | 214.0 | 201.8 |  | hsa-miR-32-5p | MIMAT0000090 | 179.8 |
| hsa-miR-411-5p | MIMAT0003329 | 184.9 | 151.1 | 280.2 | 205.4 | 67.0 |  | hsa-miR-548ad | MIMAT0018946 | 173.4 |
| hsa-miR-210 | MIMAT0000267 | 90.7 | 207.9 | 311.5 | 203.3 | 110.5 |  | hsa-miR-2116-5p | MIMAT0011160 | 172.4 |
| hsa-miR-193a-5p | MIMAT0004614 | 131.2 | 258.1 | 211.8 | 200.4 | 64.2 |  | hsa-miR-2117 | MIMAT0011162 | 172.4 |
| hsa-miR-423-5p | MIMAT0004748 | 188.5 | 176.2 | 218.5 | 194.4 | 21.8 |  | hsa-miR-28-5p | MIMAT0000085 | 171.3 |
| hsa-miR-152 | MIMAT0000438 | 164.6 | 168.8 | 243.9 | 192.4 | 44.6 |  | hsa-miR-302d-3p | MIMAT0000718 | 171.3 |
| hsa-miR-26b-5p | MIMAT0000083 | 177.7 | 174.4 | 221.9 | 191.3 | 26.5 |  | hsa-miR-378a-3p+378i | MIMAT0000732 | 170.3 |
| hsa-miR-28-5p | MIMAT0000085 | 120.5 | 175.3 | 269.2 | 188.3 | 75.2 |  | hsa-miR-548ah-5p | MIMAT0018972 | 169.2 |
| hsa-miR-495 | MIMAT0002817 | 156.3 | 146.5 | 242.2 | 181.6 | 52.7 |  | hsa-miR-1246 | MIMAT0005898 | 167.1 |
| hsa-miR-148a-3p | MIMAT0000243 | 202.8 | 12.4 | 319.1 | 178.1 | 154.8 |  | hsa-miR-4531 | MIMAT0019070 | 163.9 |
| hsa-miR-151a-3p | MIMAT0000757 | 118.1 | 196.7 | 201.6 | 172.1 | 46.9 |  | hsa-miR-331-5p | MIMAT0004700 | 161.8 |
| hsa-miR-127-3p | MIMAT0000446 | 161.0 | 161.3 | 185.5 | 169.3 | 14.1 |  | hsa-miR-126-3p | MIMAT0000445 | 159.7 |
| hsa-miR-331-3p | MIMAT0000760 | 87.1 | 217.2 | 202.5 | 168.9 | 71.3 |  | hsa-miR-183-5p | MIMAT0000261 | 158.7 |
| hsa-miR-197-3p | MIMAT0000227 | 133.6 | 168.8 | 199.9 | 167.4 | 33.2 |  | hsa-miR-583 | MIMAT0003248 | 158.7 |

Supplementary Table1. Top 100 Expressed miRNAs for TAS Cell Lines and PC Cell Line L3.6pl (continue 3)

| **TAS Cells** | | | | | | |  | **PC Cells** | | |
| --- | --- | --- | --- | --- | --- | --- | --- | --- | --- | --- |
| **miRNA probe ID** | **Accession no.** | **TAS1** | **TAS2** | **TAS3** | **Mean** | **SD** |  | **miRNA probe ID** | **Accession no.** | **L3.6pl** |
| hsa-miR-197-3p | MIMAT0000227 | 133.6 | 168.8 | 199.9 | 167.4 | 33.2 |  | hsa-miR-583 | MIMAT0003248 | 158.7 |
| hsa-miR-520h | MIMAT0002867 | 158.6 | 156.7 | 176.3 | 163.9 | 10.8 |  | hsa-miR-630 | MIMAT0003299 | 156.5 |
| hsa-miR-27a-3p | MIMAT0000084 | 90.7 | 182.8 | 217.7 | 163.7 | 65.6 |  | hsa-miR-185-5p | MIMAT0000455 | 153.4 |
| hsa-miR-155-5p | MIMAT0000646 | 127.6 | 160.4 | 194.0 | 160.7 | 33.2 |  | hsa-miR-30e-5p | MIMAT0000692 | 153.4 |
| hsa-miR-4455 | MIMAT0018977 | 140.8 | 232.1 | 106.9 | 159.9 | 64.7 |  | hsa-miR-548x-3p | MIMAT0015081 | 153.4 |
